# Integrity situational judgement test for medical school selection: judging ‘what to do’ versus ‘what not to do’

**DOI:** 10.1111/medu.13498

**Published:** 2018-01-19

**Authors:** Wendy E de Leng, Karen M Stegers‐Jager, Marise Ph Born, Axel P N Themmen

**Affiliations:** ^1^ Institute of Medical Education Research Rotterdam Erasmus Medical Centre Rotterdam The Netherlands; ^2^ Department of Psychology Erasmus University Rotterdam Rotterdam The Netherlands; ^3^ Department of Internal Medicine Erasmus Medical Centre Rotterdam The Netherlands

## Abstract

**Context:**

Despite their widespread use in medical school selection, there remains a lack of clarity on exactly what situational judgement tests (SJTs) measure.

**Objectives:**

We aimed to develop an SJT that measures integrity by combining critical incident interviews (inductive approach) with an innovative deductive approach. The deductive approach guided the development of the SJT according to two established theoretical models, of which one was positively related to integrity (honesty–humility [HH]) and one was negatively related to integrity (cognitive distortions [CD]). The Integrity SJT covered desirable (HH‐based) and undesirable (CD‐based) response options. We examined the convergent and discriminant validity of the Integrity SJT and compared the validity of the HH‐based and CD‐based subscores.

**Methods:**

The Integrity SJT was administered to 402 prospective applicants at a Dutch medical school. The Integrity SJT consisted of 57 scenarios, each followed by four response options, of which two represented HH facets and two represented CD categories. Three SJT scores were computed, including a total, an HH‐based and a CD‐based score. The validity of these scores was examined according to their relationships with external integrity‐related measures (convergent validity) and self‐efficacy (discriminant validity).

**Results:**

The three SJT scores correlated significantly with all integrity‐related measures and not with self‐efficacy, indicating convergent and discriminant validity. In addition, the CD‐based SJT score correlated significantly more strongly than the HH‐based SJT score with two of the four integrity‐related measures.

**Conclusions:**

An SJT that assesses the ability to correctly recognise CD‐based response options as inappropriate (i.e. what one should *not* do) seems to have stronger convergent validity than an SJT that assesses the ability to correctly recognise HH‐based response options as appropriate (i.e. what one should do). This finding might be explained by the larger consensus on what is considered inappropriate than on what is considered appropriate in a challenging situation. It may be promising to focus an SJT on the ability to recognise what one should *not* do.

## Abstract 2

The authors demonstrate that an SJT score based on recognizing what not to do has stronger convergent validity than an SJT score based on recognizing what to do.

## Introduction

In addition to the cognitive instruments used in selection for medical school, there is an increasing need for tools that assess non‐cognitive attributes (e.g. integrity). This growing need has led to the introduction of new medical school selection instruments such as multiple mini‐interviews, selection centres, personality and emotional intelligence assessments and situational judgement tests (SJTs).^1^, ^2^ The SJT presents applicants with challenging situations they may encounter during medical school. These situations are followed by a number of possible responses for which applicants need to judge the appropriateness.^3^ Previous studies on SJTs in medical school selection demonstrated predictive validity and incremental validity over cognitive ability tests.^4^, ^5^, ^6^, ^7^ Furthermore, SJTs result in less adverse impact than traditional cognitive tests with respect to applicants with backgrounds of low socio‐economic status.^8^

The application of an SJT in medical school selection necessitates the identification of what is measured by an SJT because this high‐stakes process requires clarity on the constructs used for selection. However, few studies elaborate on exactly what SJTs measure.^9^ This limited attention can be explained by the fact that most SJTs use an inductive development approach in which the content of the SJT is matched as closely as possible to the criterion domain (e.g. job performance).^9^, ^10^ Most inductive development approaches base the content of the SJT on critical incidents (i.e. anecdotal incidents of exceptionally good and exceptionally poor behaviour).^10^, ^11^ This point‐to‐point correspondence with the criterion contributes to the perceived job‐relatedness of an SJT^4^ and the contextualisation may strengthen its predictive validity.^12^ However, the inductive development method gives little insight into which constructs are measured because the criterion domain tends to be highly heterogeneous and to consist of various technical, interpersonal and motivational aspects.^13^

By contrast, a deductive development approach bases the content of an SJT on a specific construct by using a literature review, a job analysis or an existing theory.^10^ The deductive approach has several advantages. Firstly, it facilitates better understanding of why an SJT is related or unrelated to the criterion domain.^9^ Secondly, it supports more meaningful comparisons with other predictors of future performance,^13^ which are valuable when an admission board intends to apply different weights to the various components of a selection battery.^14^ Finally, it enables the comparison of different SJT formats (e.g. written versus video‐based) designed to measure the same construct.^15^ A possible disadvantage of the deductive method is reduced realism.

To benefit from the strengths of both methods, we combined the inductive and deductive approaches to develop an SJT measuring applicants’ knowledge of appropriate and inappropriate responses to integrity‐related situations in medical school (henceforth: Integrity SJT). Integrity is considered a core competency for medical doctors across various medical specialties^16^, ^17^ and is therefore considered a relevant construct for selection. Integrity was characterised by honesty, sincerity, fairness and modesty^18^ and the absence of inaccurate self‐serving thoughts and antisocial and counterproductive behaviour.^19^ We are aware of three deductively developed SJTs to measure integrity, including two outside and one within medical education. Firstly, Becker^20^ applied a set of integrity values in developing an SJT measuring employee integrity. This SJT was associated with integrity‐related work outcomes. Secondly, de Meijer et al.^21^ developed a video‐based SJT for the Dutch police consisting of scenarios depicting police integrity violations. This SJT was related to established integrity‐related measures and unrelated to cognitive ability and thus demonstrated both convergent and discriminant validity.^21^ Finally, Husbands et al.^22^ developed an integrity SJT for medical school admission based on a literature review on integrity constructs (e.g. honesty). This SJT correlated to honesty–humility, the integrity‐related subscale of the HEXACO personality inventory.^22^ By contrast with the traditional Big Five personality model, the HEXACO personality model consists of six dimensions as a result of the addition of the honesty–humility dimension.^23^

The present study contributes to the existing research on two points. Firstly, we developed an SJT that covers appropriate and inappropriate responses. In this way, the SJT assesses the ability to identify appropriate responses, as well as the ability to identify inappropriate responses. We distinguished these two abilities because previous researchers suggested that they involve different skills.^24^ Secondly, we used an innovative deductive development approach to create the desirable and undesirable response options in the SJT whereby two established theoretical models (one positively and one negatively related to integrity) were used to guide the development of the response options. The deductive approach was combined with an inductive approach (i.e. critical incident interviews) to ensure the realism of the SJT. Next, we addressed the research question: What are the convergent and discriminant validity levels of the Integrity SJT? Convergent validity was examined according to the relationship with external integrity‐related measures. Discriminant validity was investigated using the relationship with an unrelated external measure (i.e. self‐efficacy). The validity levels of scores based on the appropriate and inappropriate response options of the SJT were compared. With the combination of the inductive and the innovative deductive development approach, we aimed to enhance the convergent and discriminant validity of an SJT measuring integrity. In addition, we aimed to investigate the effect of the distinction between ‘what to do’ and ‘what not to do’ on the construct validity of the Integrity SJT. The outcomes of this study will add to the knowledge about this increasingly popular tool in medical school selection.

## **Methods**

### Context

This study was conducted at the Erasmus Medical Centre (MC) Medical School, Rotterdam, the Netherlands. In the Netherlands, all entry to medical school is predominantly at the undergraduate level. Admission to the Erasmus MC Medical School at the time of the study was based on three aspects: pre‐university grade point average; extracurricular activities (e.g. work‐related activities in health care), and performance on five cognitive study skill tests (e.g. scientific reading) administered during three testing days.^25^ The SJT was not part of the admission procedure but was administered solely for research purposes. Approximately 50% of the applicants were admitted to the Erasmus MC Medical School.

Six months before the testing days, the Erasmus MC Medical School organised a selection orientation day to inform medical school applicants about the selection process. Participation in the selection orientation day was voluntary and free of charge.

### Participants and procedure

The Integrity SJT was administered to the 402 participants at the 2015 selection orientation day. Participation in the SJT was voluntary. Participants were informed about the purpose of the administration and that their answers would not influence the admission decision. Informed consent was obtained from all participants. The data in this study were confidentially processed. The pencil‐and‐paper administration took place in a lecture hall at the Erasmus MC Medical School campus. The Ethics Committee of the Institute of Psychology, Erasmus University Rotterdam, deemed this study to have no need for further ethical approval by the Medical Ethics Committee.

### Measures

#### Demographic questionnaire

A demographic questionnaire was administered to determine the participants’ ethnic and socio‐economic backgrounds. An individual was classified as belonging to an ethnic minority if at least one of his or her parents had been born outside the Netherlands (i.e. the definition used by Statistics Netherlands^26^). Otherwise, an individual was classified as Dutch. Socio‐economic background was determined according to the level of education of the participants’ parents. First‐generation university students are individuals whose parents did not attend higher education.^27^

#### Development of the Integrity SJT

The deductive development approach was guided by two integrity‐related models: the honesty–humility (HH) subscale of the HEXACO personality inventory, and the How I Think questionnaire measuring cognitive distortions (CDs). The HH dimension has been demonstrated to be positively related to integrity^28^ and was used to create desirable responses. The CDs describe inaccurate thinking styles which may lead to antisocial behaviours^19^ that are negatively associated with integrity.^29^ Therefore, these were used to create undesirable responses. Specifically, sets of response options were written to represent each of seven response option categories assembled according to three HH facets (i.e. sincerity, fairness and modesty) and four CD categories (i.e. self‐centredness, blaming others, minimising and assuming the worst). These response option categories are described in Table 1.

**Table 1:** Short description of each response option category including the number of items per category for both versions of the situational judgement test

		Version	
Response option category	Short description	A	B
Honesty–humility facet			
Sincerity	Being honest and genuine	20	18
Fairness	Being fraud‐ and corruption‐avoidant	20	18
Greed avoidance	Being unmaterialistic	–	–
Modesty	Not claiming special treatment	18	20
Cognitive distortion category			
Self‐centredness	Putting one's own needs and desires above those of others (egocentrism)	15	15
Blaming others	Misattributing antisocial behaviour to outside sources	14	13
Minimising/mislabelling	Regarding antisocial behaviour as harmless/using dehumanising labels on others	15	14
Assuming the worst	Interpreting antisocial behaviour as a reaction to hostile intentions attributed to others	14	14

Greed avoidance and Mislabelling were not used for the SJT in this study.

The inductive development approach consisted of critical incident interviews with nine subject matter experts (SMEs), who were individuals directly involved in the assessment of professional behaviour of medical students (e.g. clinical skills teachers). These SMEs described incidents in which a medical student behaved unprofessionally (e.g. by cheating). Further questions were asked to provide elaboration on these critical incidents following the technique described by Flanagan.^11^ These incidents formed the basis of the SJT scenarios. The scenarios were presented to a group of medical students and staff (*n* = 41) to gather input for realistic response options. To stimulate the development of response options, scenarios were presented with a number of prompts (e.g. What would be the best/worst/most likely response to this situation?).^30^

The resulting Integrity SJT consisted of 57 scenarios. This pilot version of the Integrity SJT was randomly split into two versions (i.e. Version A and Version B) because of the large number of scenarios. Each scenario was followed by four response options, of which two represented HH facets and two represented CD categories. Table 1 presents the distribution of items across the seven response option categories. All scenarios described situations at the beginning of medical school. No medical knowledge was required to understand the scenarios because the target population of this study were applicants for undergraduate entry who, in general, have limited experience in health care. On average, scenarios were described in 56.4 words and response options in 12.9 words. An example SJT item is given in Box 1. Five additional example items are presented in Table S1, online.

Box 1 **Example scenario (including corresponding response option categories)**

**Table:** 

John finds out that Mary has a copy of the examination paper that will be given next week. She tells him that she has already sold the examination paper to some fellow students and asks him if he also wants to look at the paper							
*Judge each of the following response options on how appropriate they would be for John*							
		**Very inappropriate**			**Very appropriate**		
1	Look at the examination paper because everyone would do that (Minimising)	1	2	3	4	5	6
2	Don't look at the examination paper because you are not entitled to do so (Modesty)	1	2	3	4	5	6
3	Look at the examination paper and tell no‐one you did (Self‐centredness)	1	2	3	4	5	6
4	Don't look at the examination paper and inform the teacher (Fairness)	1	2	3	4	5	6

Each SJT item was scored by calculating the squared distance between a participant's judgement and the average judgement across all other participants. To ensure that the SJT score was not influenced by responder tendencies to use the rating scale in a certain manner (e.g. extreme response style), this calculation was preceded by a within‐person *Z* standardisation so each participant had a mean score of 0 and a standard deviation (SD) of 1.^31^ Unlike most SJTs, SMEs did not contribute to the scoring key as previous research has demonstrated the similarity of judgements of novices and experts.^32^, ^33^, ^34^ However, to guarantee the comparability of novices and experts in this study, we compared item scores based on the average judgement of the group of participants with item scores based on the average judgement of a group of general practice (GP) residents (*n* = 63). These residents were chosen as a reference group because this group includes a relatively large number of residents who are trained as generalists. For the GP residents, the SJT was split into three versions of 19 scenarios (*n*_I_ = 23, *n*_II_ = 18, *n*_III_ = 22) in order to reduce the time investment. The mean ± SD age of the GP residents was 28.6 ± 2.7 years and 52 (82.5%) of them were female. Fifty‐one (81.0%) GP residents were Dutch and 21 (33.0%) were first‐generation university students. Table S2 (online) presents the intraclass correlation coefficients for the GP residents for the total SJT score, the subscore based on the HH SJT items and the subscore based on the CD SJT items.

### Convergent and discriminant validity

Convergent validity was examined by the relationship between the Integrity SJT and the two integrity‐related measures used for assembling the response option categories: the HH subscale of the HEXACO Simplified Personality Inventory (HEXACO‐SPI)^35^ and the How I Think (HIT) questionnaire measuring CDs.^19^, ^36^ To thoroughly analyse the convergent validity, we examined the relationship with two additional integrity‐related measures: the student‐related items of the Inventory of Counterproductive Behaviour (ICB)^37^, ^38^ and the workplace deviance measure.^39^ The student‐related items of the ICB assess counterproductive academic behaviour (i.e. intentional behaviours in conflict with the objectives of an educational institution).^40^ Workplace deviance refers to the deliberate violation of the norms of an organisation.^41^ The items of the workplace deviance measure were rewritten to fit the context and two items were deleted because they were considered irrelevant to an academic context.

Discriminant validity was examined according to the relationship with the self‐efficacy subscale of the Motivated Strategies for Learning Questionnaire (MSLQ).^42^ Self‐efficacy is a person's belief in his or her ability to reach desired goals.^43^ Self‐efficacy is an important predictor of medical school performance,^44^, ^45^ but is expected to be unrelated to integrity. The items were slightly adapted to fit the context of the study. The characteristics of these measures are described in Table S3, online.

### Statistical analyses

Three SJT scores were computed by adding up scores across: (i) all items (i.e. total SJT score); (ii) all HH‐based items, and (iii) all CD‐based items. Scores were reversed so that higher scores indicated better performance on the Integrity SJT. Pearson's correlation coefficients were calculated between the three SJT scores and the integrity‐related measures and self‐efficacy subscale. The correlation coefficients were merged across the two versions of the Integrity SJT using a random‐effects meta‐analytic approach. The difference between the HH‐based and CD‐based SJT scores in their correlations with the integrity‐related measures was analysed with the Williams’ test.^46^ Given the large number of correlations, a stricter alpha level was used (α = 0.01). Correlation analyses were conducted using IBM spss Statistics for Windows Version 21.0 (IBM Corp., Armonk, NY, USA). r Version 3.1.0 (http://www.R-project.org) was used to meta‐analytically merge the correlation coefficients (‘metacor’ package) and to conduct the Williams’ test (‘psych’ package).

## **Results**

### Demographics

The numbers of participants completing Versions A and B of the SJT were 186 (response rate: 92.5%) and 181 (response rate: 90.0%), respectively. There were no significant differences in age, gender, ethnicity or socio‐economic background between participants completing Versions A and B (Table2 ). The mean age of the undergraduate entry applicants was 17.8 years, 271 participants were female (73.8%), 132 came from ethnic minorities (36.0%) and 108 were first‐generation university students (29.4%). Scores on the integrity‐related measures and self‐efficacy subscale were comparable for the participants of the two versions, except for the HIT questionnaire (*t*_(354)_ = − 2.77, p = 0.006, *d* = 0.29). However, the effect size of this difference was small and both groups scored well below the average score of a normative sample of 412 youths (mean score: 2.39).^36^ Of the participants in the selection orientation day, 352 applied to medical school (87.6%), indicating that the participants were suitably representative of medical school applicants. For both Versions A and B, examination of the skewness and kurtosis of the SJT score distributions showed negative skewness (Table2) (i.e. most participants obtained a high score on the SJT).

**Table 2:** Respondent demographics and descriptive data for the study's measures

	Version A (*n* = 186)	Version B (*n* = 181)	Range (min–max)
Gender: female, %	75.1%	72.9%	
Age, years, mean ± SD	17.8 ± 2.2	17.7 ± 1.8	
Ethnicity: Dutch, %	63.4%	63.9%	
First‐generation university, %	28.1%	31.1%	
Integrity‐related measures			
HEXACO‐SPI honesty–humility	43.36 (6.01)	44.36 (6.06)	16–80
HIT questionnaire	**1.63 (0.42)**	**1.75 (0.41)**	1–6
ICB student‐related items	2.89 (0.84)	2.82 (0.91)	1–6
Workplace deviance measure	2.38 (0.81)	2.23 (0.86)	1–7
MSLQ self‐efficacy subscale	46.27 (5.76)	45.33 (6.64)	8–56
Skewness			
Total	−1.97	−1.83	
HH‐based	−2.03	−1.62	
CD‐based	−1.59	−1.94	
Kurtosis			
Total	4.09	3.67	
HH‐based	4.65	2.48	
CD‐based	2.31	4.37	

CD = cognitive distortions; HEXACO‐SPI = HEXACO Simplified Personality Inventory; HH = honesty–humility; HIT = How I Think; ICB = Inventory of Counterproductive Behaviour; MSLQ = Motivated Strategies of Learning Questionnaire; SD = standard deviation.

Bold numbers indicate a significant difference (p < 0.01, two‐tailed).

### Preliminary analyses

For each SJT item, two scores were generated: one of these used the GP residents as a reference and the other used the group of participants itself as a reference. Correlations between these two scores were calculated. For Version A, the average correlation across the 116 items was 0.93 (range: 0.27–1.00). All but three items had a correlation above 0.50 (i.e. large effect size^47^). For Version B, the average correlation across the 112 items was 0.93 (range: 0.11–1.00). Only two items had a correlation below 0.50. The negligible number of correlations below 0.50 was deemed sufficient to confirm the use of a scoring key with the group of participants itself as a reference.

### Main analyses

All SJT scores (i.e. total, HH‐based and CD‐based) correlated significantly with the four external integrity‐related measures (Table3). The correlations were in the expected direction and indicated a moderate effect size (−0.22 ≤ *r* ≤ 0.40). Appendix S1 (online) presents the correlations between the individual response option categories, HH facets and CD categories.

**Table 3:** Descriptive data for the total score, the honesty–humility (HH)‐based and cognitive distortions (CD)‐based situational judgement test (SJT) scores and correlations between total score, HH‐based and CD‐based SJT scores and the integrity‐related measures and self‐efficacy subscale

SJT score	Version A		Version B		Integrity‐related measures (95% CI)				SE
	M/max	SD	M/max	SD	HH	HIT^a^	ICB^a^	WD	
Total	82.44	19.13	77.77	22.85	**0.37** (0.27 to –0.45)	**−0.35** (−0.50 to −0.17)	**−0.34** (−0.55 to −0.09)	**−0.27** (−0.40 to −0.13)	0.01 (−0.10 to 0.11)
HH‐based	75.45	10.15	80.79	12.43	**0.29** (0.19 to –0.38)	**−0.26** (−0.41 to −0.13)	**−0.26** (−0.44 to −0.06)	**−0.22** (−0.32 to −0.11)	−0.01 (−0.11 to 0.10)
CD‐based	81.21	10.08	81.44	11.68	**0.40** (0.31 to –0.49)	**−0.40** (−0.58 to −0.18)	**−0.38** (−0.60 to −0.10)	**−0.29** (−0.44 to −0.13)	0.02 (−0.08 to 0.13)

CI = confidence interval; HIT = How I Think; ICB = Inventory of Counterproductive Behaviour; M/max = mean as a percentage of the maximum score (because Versions A and B have different numbers of items); SD = standard deviation; SE = self‐efficacy; WD = workplace deviance.

Descriptive data are presented for each version separately; correlations are meta‐analytically merged across both versions.

Bold coefficients depict a significant correlation (p < 0.01, two‐tailed).

a Integrity‐related measures with a significantly different correlation with the CD‐based SJT score than the HH‐based SJT score (p < 0.01).

All correlation coefficients with the integrity‐related measures were – in absolute terms – larger for the CD‐based SJT score than for the HH‐based SJT score (Table3). The Williams’ test indicated that the CD‐based SJT score correlated significantly more strongly than the HH‐based SJT score with the HIT questionnaire (*t*_(168)_ = 3.07, p = 0.003, *d* = 0.47) and with the ICB (*t*_(171)_ = 2.69, p = 0.008, *d* = 0.41). The CD‐based SJT score correlated more strongly than the HH‐based SJT score with the honesty–humility subscale, but this difference was only marginally significant (*t*_(173)_ =−2.54, p = 0.011, *d* = 0.39). No significant difference was found between the HH‐based and CD‐based SJT scores in their correlation with the workplace deviance measure (*t*_(169)_ = 1.50, p = 0.130).

As expected, none of the SJT scores were significantly correlated to the self‐efficacy subscale (Table3).

## **Discussion**

The results of this study indicate that the Integrity SJT had convergent and discriminant validity. This is evidenced by a significant correlation with integrity‐related measures and no correlation with a self‐efficacy subscale. Additionally, the findings indicate that an SJT score representing CD categories has stronger convergent validity than an SJT score representing HH facets. This is demonstrated by significantly higher correlations with two of the four integrity‐related measures for the CD‐based SJT score than for the HH‐based SJT score.

The first finding implies that the use of a deductive development approach based on established theoretical models together with a traditional inductive approach generates an SJT that has convergent validity. The correlation with the HH subscale found in this study appears to be somewhat stronger than the correlation coefficient reported in the study by de Meijer et al.^21^ and is similar to the uncorrected correlation coefficient reported in the study by Husbands et al.^22^ The strength of the correlation with the HIT questionnaire found in this study is similar to that of the correlation reported in the study by de Meijer et al.^21^ However, a prior study demonstrated a negative association between the score on the HIT questionnaire and a person's level of education.^48^ Thus, the correlation with the HIT questionnaire in this study might be attenuated by the high pre‐university education level of the participants. Different SJTs and contexts in these studies make it difficult to perform a direct comparison of the correlation coefficients. Nonetheless, the established integrity‐related models proved to be a useful guide to deductively develop the Integrity SJT. Moreover, the convergent validity of the Integrity SJT was at least as strong as the correlations reported in prior studies.^21^, ^22^ The use of theoretical models for the development of an SJT is supported by previous studies on SJTs outside the medical domain measuring constructs other than integrity. For example, an SJT developed on the basis of eight dimensions of an existing leadership model was significantly correlated to an external leadership questionnaire.^49^ Additionally, an SJT developed on the basis of a conflict management model was significantly related to supervisor ratings of on‐the‐job conflict management.^50^ Overall, these findings suggest that a deductive development approach based on established theoretical models enhances the construct and predictive validity of an SJT. Future research is required to identify which characteristics of the deductive development approach positively influence the SJT's validity and should attempt to make a more direct comparison of the two development approaches. The positive findings with respect to the use of theoretical models in SJT development should not diminish the importance of the inductive development approach. The inductive approach uses empirical data to contextualise the SJT's content. The contextualisation could lead to stronger predictive validity,^51^ higher perceived job‐relatedness^52^ and lower susceptibility to socially desirable responding than, for example, non‐contextualised personality tests.^53^ The strengths of an SJT are enhanced by a combination of both development methods.

The second finding of this study indicates that an SJT score based on the ability to identify what one should *not* do has stronger convergent validity than an SJT score based on the ability to identify what one should do. This finding is in line with that in a prior study on sales and management SJTs, which demonstrated stronger predictive validity for the ability to identify the worst response option than for the ability to identify the best response option.^24^ A similar finding was reported in another SJT study on teachers’ tacit knowledge in which a subscale assessing the ability to detect bad responses was better able to discriminate experts from novices than a subscale assessing the ability to detect good responses.^54^ This finding might be explained by a larger consensus on what is considered inappropriate than on what is considered appropriate in a challenging situation. There exist a variety of reactions that may be considered appropriate but the eventual response depends on the type of job, organisation and culture (e.g. appropriately solving a problem with one's supervisor differs between vertical and horizontal organisational structures). However, inappropriate reactions are most likely to always lead to negative outcomes regardless of the type of job, organisation or culture.^24^ Indeed, the GP residents in this study showed greater agreement in their judgements of the CD‐based response options than in their judgements of the HH‐based response options. Unlike prior studies that empirically determined the best and worst responses (e.g. using SMEs),^20^, ^45^ the present study deductively established desirable and undesirable responses. The deductive development approach does not require the input of SMEs, which may be beneficial because it can be difficult to determine who is best placed to serve as an expert and practically inconvenient to collect data from this group. However, we have not yet examined the relationship of the Integrity SJT with future performance and therefore further research is necessary to determine if the stronger predictive validity for the ability to identify what one should *not* do is also observed for the SJT in this study.

### Strengths, limitations and recommendations for future research

An important strength of this study lies in its combination of two development approaches, which allows us to benefit from the advantages of both methods and results in an SJT with realistic contextualised scenarios measuring an explicit construct. A second strength is the large number of integrity‐related measures used in this study, which supports the credibility of our statements regarding convergent validity. A third strength refers to the fact that, unlike most previous studies, the current work not only examined convergent validity, but also investigated discriminant validity, thereby indicating that the Integrity SJT is associated with theoretically related constructs and not associated with theoretically unrelated constructs.

Despite its strengths, this study has some limitations. Firstly, the response options of the Integrity SJT were written to represent response option categories by aligning the wording and reasoning of response options belonging to the same category. Future research might improve the accuracy of this categorisation by performing an additional classification by an independent group. Secondly, the assumption that the HH facets reflect good responses and that CDs reflect bad responses may be too simplistic. For example, an HH‐based response might entail the betrayal of one's friend and a CD‐based response might seem to be made inevitable by group pressure. The influence of these subtleties on the functioning of an SJT should be further investigated. Thirdly, the investigation of systematic ethnic differences in the score on the Integrity SJT was beyond the scope of this paper, but future research is necessary to examine the ‘what to do’ versus ‘what not to do’ distinction with regard to adverse impact. Fourthly, critical incident interviews were conducted with only nine SMEs. Although the critical incident interviews produced a wealth of data, interviews with more SMEs may have led to a wider coverage of the professional issues encountered by medical students. Finally, the results of this study are derived solely from its administration within an admission context with undergraduate entry. As a result, the patient‐centredness of the SJT scenarios was limited, which may reduce the generalisability of the present results to SJTs used for graduate entry into medical school. Although the Integrity SJT involved some patient‐related scenarios, future research should investigate the generalisability of this study's findings to other settings.

These findings elicit the following recommendations for future research. Firstly, the Integrity SJT showed stronger convergent validity for the CD‐based score than for the HH‐based score. However, it is possible that for other constructs (e.g. empathy), a score based on the correct identification of desirable responses will have stronger convergent validity than a score based on the correct identification of undesirable responses, perhaps because desirable responses are more obvious for certain constructs. Future research is necessary on the generalisability of the CD‐based score's stronger convergent validity to SJTs measuring other constructs. Finally, future research on the predictive validity is a necessary requirement before an SJT can be considered for inclusion in medical school selection.

### Practical implications

A first practical implication for medical schools using or planning to use a construct‐based SJT in their selection procedures is the use of established theoretical models to guide the deductive development of an SJT. The theoretical models may be related to integrity, but may also involve other constructs (e.g. social competence).

A second practical implication is that an SJT might be used to assess the ability to correctly identify what one should *not* do in a challenging situation. This implication could support the proposal to use an SJT for screening out medical school applicants^2^ as SJTs appear to be more informative at the lower end of the distribution.^55^, ^56^ Only a small group of medical students behaves unprofessionally and is unresponsive to remediation activities as a result of poor insight and poor adaptability.^57^ An SJT that assesses the ability to identify inappropriate response options may improve the ability to accurately identify unsuitable applicants. The application of an SJT as a screen‐out test must take into account the high base rate of suitable applicants^58^ and the low prevalence of unprofessional behaviour.^59^ Future research to indicate the precise use of the SJT in medical selection procedures is necessary.

## **Conclusions**

The combination of a traditional inductive and an innovative deductive development approach resulted in an Integrity SJT which had convergent and discriminant validity. Categorising the response options of the SJT according to two established theoretical models – one positively and one negatively related to integrity – resulted in a wide range of appropriate (HH‐based) and inappropriate (CD‐based) response options. The CD‐based SJT score had stronger convergent validity than the HH‐based SJT score. It may be promising to focus SJTs on the ability to correctly identify inappropriate response options (i.e. what one should *not* do).

## Contributors

WEdL and KMS‐J conducted the critical incident interviews in this study. All authors contributed to the development of the Integrity situational judgement test (Integrity SJT). WEdL and APNT collected the data for the validation of the Integrity SJT. WEdL analysed the data and wrote the first draft of the paper. All authors contributed to the interpretation of the statistical analyses and the critical revision of the paper. All authors approved the final manuscript for publication and are accountable for this work.

## Funding

None.

## Conflicts of interest

None.

## Ethical approval

The study design was reviewed by the independent Ethical Committee of the Institute of Psychology, Erasmus University Rotterdam, which concluded that no further ethical approval by the Medical Ethics Committee was warranted.

## Supporting information


**Table S1**. Five example scenarios used in the integrity situational judgement test.

**Table S2**. Intraclass correlation coefficients for general practice residents for the total situational judgement test (SJT) score and the subscores based on the honesty–humility items and cognitive distortion items.

**Table S3**. Characteristics of the measures used to assess convergent and discriminant validity.

**Appendix S1**. Correlations between the individual response option categories, honesty–humility facets and cognitive distortion categories.
