# Supplementary material for: Integrity situational judgement test for medical school selection: judging ‘what to do’ versus ‘what not to do’
Source: Med Educ. 2018 Jan 19;52(4):427–37. doi: 10.1111/medu.13498 (PMC5901405; doi:10.1111/medu.13498)
Supplement: Supplementary file 1 — Table S1. Five example scenarios used in the integrity situational judgement test. [file MEDU-52-427-s001.docx]

| **Scenario 1** | | |
| --- | --- | --- |
| Michelle, Tim and Sarah have been given a group assignment. The group meets for the first time to discuss how to approach the assignment. During this meeting, it becomes clear that Sarah has already completed the assignment. Sarah explains that she had some spare time in which she completed the group assignment, but that she doesn’t mind to share her work with Michelle and Tim. Tim says he is satisfied with this deal.  *Judge for each of the following response options how appropriate they would be for Michelle.* | | |
|  |  | Very Very  inappropriate appropriate |
| 1. | Accept Sarah’s offer because that is the most easy thing to do in this case. | 1 2 3 4 5 6 |
| 2. | Accept Sarah’s offer because it would be a waste to carry out the same assignment twice. | 1 2 3 4 5 6 |
| 3. | Thank Sarah for the offer but propose to split up the assignment among themselves. | 1 2 3 4 5 6 |
| 4. | Together with Tim, complement Sarah’s work to ensure your contribution to the group assignment. | 1 2 3 4 5 6 |

| **Scenario 2** | | |
| --- | --- | --- |
| Stacey overhears two fellow students talk about yesterday’s clinical skills course. During this course, students practice physical examinations on each other. The two students gossip about Maarten, a fellow student who has a neaves (a birth mark) on a large part of his body.  *Judge for each of the following response options how appropriate they would be for Stacey.* | | |
|  |  | Very Very  inappropriate appropriate |
| 1. | Act as if she does not hear her fellow students because she doesn’t care. | 1 2 3 4 5 6 |
| 2. | Tell her fellow students that she can hear them gossiping about Maarten. | 1 2 3 4 5 6 |
| 3. | Tell her fellow students that they should stop gossiping because they are violating the confidentiality rules. | 1 2 3 4 5 6 |
| 4. | Say nothing because her fellow students would view her as a bore if she did. | 1 2 3 4 5 6 |

| **Scenario 3** | | |
| --- | --- | --- |
| Eva finds out that she has to hand in an essay tomorrow. Eva forgot to note the deadline in her calendar and has not yet started with the assignment. The essay is an important part as this has to be graded as ‘sufficient’ to pass the course.  *Judge for each of the following response options how appropriate they would be for Eva.* | | |
|  |  | Very Very  inappropriate appropriate |
| 1. | Email her teacher to tell honestly that she forgot the deadline and ask for a postponement. | 1 2 3 4 5 6 |
| 2. | Resit the essay, because she should have written down the assignment in her calendar. | 1 2 3 4 5 6 |
| 3. | Make sure to have something on paper and hope for the best. | 1 2 3 4 5 6 |
| 4. | Ask for a postponement because the teacher had not posted a reminder of the deadline. | 1 2 3 4 5 6 |

| **Scenario 4** | | |
| --- | --- | --- |
| Steven and Robin are close friends. They are taking an exam. When Steven looks up he notices that Robin is using a smartphone which he has hidden in the sleeve of his sweater.  *Judge for each of the following response options how appropriate they would be for Steven.* | | |
|  |  | Very Very  inappropriate appropriate |
| 1. | Do nothing since it is the proctors’ responsibility to monitor the exam. | 1 2 3 4 5 6 |
| 2. | Meet Robin after the exam and tell him that you saw him using his smartphone. | 1 2 3 4 5 6 |
| 3. | Inform the proctors that Robin is violating the rules. | 1 2 3 4 5 6 |
| 4. | Do nothing because we all cheat sometimes. | 1 2 3 4 5 6 |

| **Scenario 5** | | |
| --- | --- | --- |
| Farid has started medical school. He joined a student association because he is new in town. The student association organizes a welcome week for new members. This welcome week, however, overlaps with a few mandatory lectures.  *Judge for each of the following response options how appropriate they would be for Farid.* | | |
|  |  | Very Very  inappropriate appropriate |
| 1. | Explain the situation to the teacher and ask if he can make up for the lectures at a different moment. | 1 2 3 4 5 6 |
| 2. | Skip the mandatory lectures and go to the welcome week. | 1 2 3 4 5 6 |
| 3. | Call in sick for the lectures since other students will probably do the same. | 1 2 3 4 5 6 |
| 4. | Try to find a solution together with the student association and the medical school. | 1 2 3 4 5 6 |
