# Supplementary material for: Integrity situational judgement test for medical school selection: judging ‘what to do’ versus ‘what not to do’
Source: Med Educ. 2018 Jan 19;52(4):427–37. doi: 10.1111/medu.13498 (PMC5901405; doi:10.1111/medu.13498)
Supplement: Supplementary file 2 — Table S2. Intraclass correlation coefficients for general practice residents for the total situational judgement test (SJT) score and the subscores based on the honesty–humility items and cognitive distortion items. [file MEDU-52-427-s002.docx]

Supplemental file 2

*Intraclass correlation coefficients (ICCs) including 95% confidence interval for the GP residents on all SJT items and the HH-based and CD-based SJT items.*

| Version | Total | HH-based | CD-based |
| --- | --- | --- | --- |
| I | .73 [.66; .80] | .37 [.26; .51] | .44 [.33; .58] |
| II | .65 [.57; .73] | .28 [.19; .41] | .40 [.29; .54] |
| III | .73 [.66; .79] | .19 [.12; .30] | .35 [.25; .49] |

*Note. HH = honesty-humility CD = cognitive distortions* 95% confidence interval between square brackets
