# Supplementary material for: Integrity situational judgement test for medical school selection: judging ‘what to do’ versus ‘what not to do’
Source: Med Educ. 2018 Jan 19;52(4):427–37. doi: 10.1111/medu.13498 (PMC5901405; doi:10.1111/medu.13498)
Supplement: Supplementary file 3 — Table S3. Characteristics of the measures used to assess convergent and discriminant validity. [file MEDU-52-427-s003.docx]

Supplemental file 3

*Number of items, rating scale and previously established internal consistency reliability of the measures used to examine the construct validity.*

| Measure | # items | rating scale | *α* |
| --- | --- | --- | --- |
| HEXACO-SPI honesty-humility | 16 | 1: *strongly disagree* - 5: *strongly agree* | .78 |
| HIT questionnaire | 54 | 1: *disagree strongly* - 6: *agree strongly* | .78- .90 |
| ICB student-related items | 25 | 1: *never even considered it* - 6: *did it three or more times* | .88 |
| Workplace deviance measure | 17 | 1: *never* - 7: *daily* | .78-.81 |
| MSLQ Self-efficacy subscale | 8 | 1: *not at all true of me* - 7: *very true of me* | .93 |

*Note.* HEXACO-SPI = HEXACO Simplified Personality Inventory HIT = How I Think

ICB = Inventory Counterproductive Behaviour MSLQ = Motivated Strategies of Learning Questionnaire
