# Supplementary material for: Integrity situational judgement test for medical school selection: judging ‘what to do’ versus ‘what not to do’
Source: Med Educ. 2018 Jan 19;52(4):427–37. doi: 10.1111/medu.13498 (PMC5901405; doi:10.1111/medu.13498)
Supplement: Supplementary file 4 — Appendix S1. Correlations between the individual response option categories, honesty–humility facets and cognitive distortion categories. [file MEDU-52-427-s004.docx]

Appendix S1

*Intercorrelations between the SJT response option categories, honesty-humility facets and cognitive distortion categories.*

|  |  | Integrity SJT | | | | | | | |  | Integrity-related measures | | | | | | | |
| --- | --- | --- | --- | --- | --- | --- | --- | --- | --- | --- | --- | --- | --- | --- | --- | --- | --- | --- |
|  |  |  |  |  |  |  |  |  |  |  |  |  |  |  |  |  |  |  |
|  |  | Sc | Bo | Mi | As |  | Si | Fa | Mo |  | Sc | Bo | Mi | As |  | Si | Fa | Mo |
| Integrity SJT | Sc |  |  |  |  |  |  |  |  |  |  |  |  |  |  |  |  |  |
|  | Bo | **.63** |  |  |  |  |  |  |  |  |  |  |  |  |  |  |  |  |
|  | Mi | **.67** | **.67** |  |  |  |  |  |  |  |  |  |  |  |  |  |  |  |
|  | As | **.59** | **.56** | **.62** |  |  |  |  |  |  |  |  |  |  |  |  |  |  |
|  |  |  |  |  |  |  |  |  |  |  |  |  |  |  |  |  |  |  |
|  | Si | **.67** | **.62** | **.58** | **.57** |  |  |  |  |  |  |  |  |  |  |  |  |  |
|  | Fa | **.68** | **.67** | **.70** | **.55** |  | **.68** |  |  |  |  |  |  |  |  |  |  |  |
|  | Mo | **.60** | **.57** | **.57** | **.52** |  | **.67** | **.68** |  |  |  |  |  |  |  |  |  |  |
|  |  |  |  |  |  |  |  |  |  |  |  |  |  |  |  |  |  |  |
| Integrity-rel. measures | Sc | **-.34** | **-.35** | **-.36** | **-.37** |  | **-.24** | **-.29** | **-.23** |  | .74/.73 |  |  |  |  |  |  |  |
|  | Bo | **-.24** | **-.26** | **-.21** | -.25 |  | **-.16** | **-.17** | -.09 |  | **.68** | .75/.68 |  |  |  |  |  |  |
|  | Mi | **-.31** | **-.34** | -.31 | -.34 |  | **-.23** | **-.30** | **-.24** |  | **.72** | **.71** | .67/.66 |  |  |  |  |  |
|  | As | **-.29** | **-.30** | -.21 | -.27 |  | **-.18** | -.18 | -.18 |  | **.65** | **.69** | **.73** | .65/.65 |  |  |  |  |
|  |  |  |  |  |  |  |  |  |  |  |  |  |  |  |  |  |  |  |
|  | Si | **.31** | **.34** | **.25** | **.30** |  | **.23** | **.25** | **.23** |  | **-.41** | **-.30** | -.37 | -.25 |  | .63/.63 |  |  |
|  | Fa | **.26** | **.31** | **.28** | **.31** |  | **.18** | .24 | **.16** |  | **-.59** | **-.51** | **-.54** | **-.47** |  | **.51** | .58/.56 |  |
|  | Mo | **.17** | **.22** | **.22** | **.12** |  | **.18** | **.17** | **.15** |  | **-.41** | **-.27** | **-.38** | **-.33** |  | **.32** | **.36** | .48/.45 |

*Note.* Sc = Self-centeredness Bo = Blaming others Mi = Minimizing As = Assuming the Worst Si = Sincerity Fa = Fairness Mo = Modesty Correlation coefficients are meta-analytically merged across version A and B Bold coefficients represent significant correlations (p <.01, two-tailed) Internal consistency reliabilities are on the diagonal line (version A/version B)
